# Low Frequency Severe-Intensity Interval Training Markedly Alters Respiratory Compensation Point During Incremental Exercise in Untrained Male

**DOI:** 10.3389/fphys.2020.01100

**Published:** 2020-09-04

**Authors:** Hidehiro Nakahara, Shin-ya Ueda, Tadayoshi Miyamoto

**Affiliations:** ^1^Graduate School of Health Sciences, Morinomiya University of Medical Sciences, Osaka, Japan; ^2^Department of Physical Education, Faculty of Education, Gifu University, Gifu, Japan; ^3^Graduate School of Human Environment, Faculty of Sport and Health Sciences, Osaka Sangyo University, Osaka, Japan

**Keywords:** second ventilatory threshold, once weekly training, high intensity exercise, respiratory adaptations, incremental maximal exercise test

## Abstract

This study investigated the effect of low-frequency severe-intensity interval training on the respiratory compensation point (RCP) during incremental exercise test. Eighteen healthy males (age; 20.7 ± 2.2 years, range 18 to 29 years, height; 174.0 ± 5.6 cm, weight; 68.8 ± 13.5 kg) were randomly assigned to an interval training group or a control group. Interval training was conducted once weekly for 3 months. Each session consisted of three bouts of bicycle ergometer exercise at 80% maximum work rate until volitional fatigue. Before (baseline) and after the 3-month intervention, incremental exercise test was performed on a bicycle ergometer for determination of ventilatory threshold (VT), RCP, and peak oxygen consumption (V̇_O__2_ peak). The training program resulted in significant increases of V̇_O__2_ peak (+ 14%, *p* < 0.001, $\eta_{\text{p}}^{2}$ = 0.437), oxygen consumption (V̇_O__2_) at VT (+ 18%, *p* < 0.001, $\eta_{\text{p}}^{2}$ = 0.749) and RCP (+ 15%, *p* = 0.03, $\eta_{\text{p}}^{2}$ = 0.239) during incremental exercise test in the training group. Furthermore, a significant positive correlation was observed between the increase in V̇_O__2_ peak and increase in V̇_O__2_ at RCP after intervention (*r* = 0.87, *p* = 0.002) in the training group. Tidal volumes at VT (*p* = 0.04, $\eta_{\text{p}}^{2}$ = 0.270) and RCP (*p* = 0.01, $\eta_{\text{p}}^{2}$ = 0.370) also increased significantly after intervention compared to baseline. Low-frequency severe-intensity interval training induced a shift in RCP toward higher work rate accompanied by higher tidal volume during incremental exercise test.

## Introduction

**FIGURE 1:**
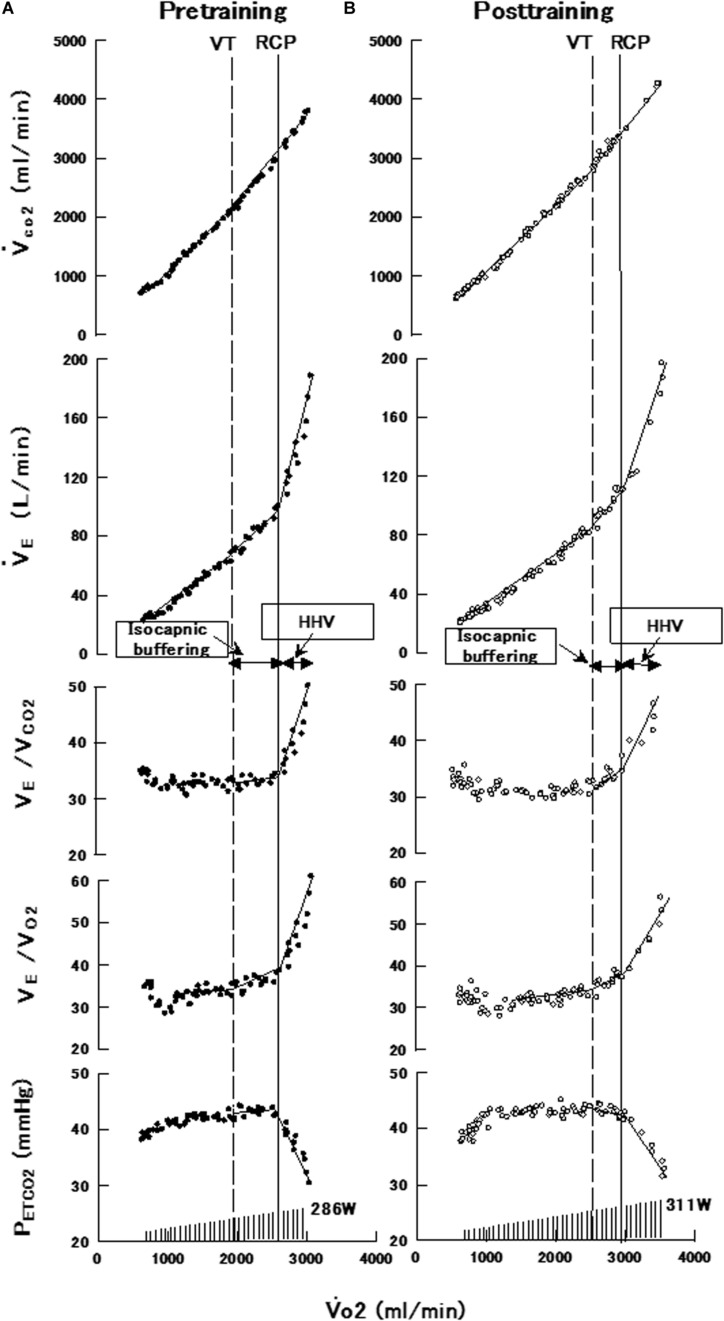
Data of a representative subject in the interval training group showing breath-by-breath measurements of V̇_CO__2_, V̇_E_, V_E_/V_CO__2_, V_E_/V_O__2_, and PET_CO__2_ against V̇_O__2_ during a progressively increasing maximal exercise test before **(A)** and after **(B)** the interval training program. V̇_O__2_, oxygen uptake; V̇_CO__2_, CO_2_ output, V̇_E_, minute ventilation; VT, ventilatory threshold; PET_CO__2_, end-tidal pressures for CO_2_; RCP, respiratory compensation point; HHV, hypocapnic hyperventilation.

Determining peak oxygen consumption (V̇_O__2_ peak), ventilatory threshold (VT) and respiratory compensation point (RCP: second ventilatory threshold) is essential for coaches and physical trainers, because they can use these physiological points as a references to establish individual training zones and to evaluate training interventions (Billat et al., 2003; Keir et al., 2015). On the other hand, RCP may be a key indicator for evaluating the limits of tolerable endurance exercise. Briefly, during incremental exercise test, minute ventilation (V̇_E_) increases in proportion to the increase in CO_2_ output (V̇_CO__2_), when bicarbonate buffering of lactic acid (metabolic acidosis) is added to metabolic CO_2_ production. The period of compensation for metabolic acidosis is called isocapnic buffering (Whipp et al., 1989). Beyond the period of isocapnic buffering, V̇_E_ increases more rapidly than V̇_CO__2_ increase, and the threshold marks the onset of hyperventilation as work rate is further increased. The point of onset of rapid V̇_E_ increase is termed RCP (Figure 1) (Whipp et al., 1989; Wasserman et al., 1994; Oshima et al., 1997, 1998). Consequently, evaluation of RCP is considered to be an important assessment of exercise tolerance to severe-intensity intermittent activities such as soccer and basketball, which consist of a combination of aerobic and anaerobic exercise, and the effectiveness of the training program (Green et al., 2003; Maciejczyk et al., 2014a,b; Keir et al., 2015).

Maciejczyk et al. (2014b) reported that RCP measurement is a better method than VT for determining high-intensity exercise performance because exceeding RCP induced much greater changes in body functions, such as an increase in lactate concentration and a development of metabolic acidosis leading to physical fatigue. They suggested that compared with VT and V̇_O__2_ peak, evaluation of RCP during incremental exercise test is an easy and better method of assessing aerobic capacity in subjects with lowered physical capacity and motivational problems, because RCP evaluation does not require performing maximum effort. In a cross-sectional study, Green et al. (2003) also reported that RCP expressed as a percentage of V̇_O__2_ peak was significantly lower in untrained subjects than in aerobic and anaerobic competitors. In a longitudinal study of young women, Ciolac et al. (2011) observed that subjects who underwent high intensity interval training for three times a week for 16 week achieved improvements of maximal oxygen uptake and oxygen uptake at RCP. We also found that 6-month endurance training of at least 6 days a week undertaken by middle-distance runners induced not only increases in V̇_O__2_ peak and VT, but also a shift in RCP toward higher work load and an improvement in the range of isocapnic buffering (Oshima et al., 1998). Furthermore, our laboratory recently demonstrated that severe-intensity interval training at a frequency of once a week for 3 months improved V̇_O__2_ peak and VT and also markedly decreased V̇_E_ and blood lactate concentration in high-intensity constant load exercise test, suggesting that the training program induced ventilatory depression during high-intensity exercise (Nakahara et al., 2015). However, the improvement of RCP after once a week and low-volume interval training is not clear, despite being a good indicator of aerobic capacity and exercise tolerance at high intensity exercise. Studies of the effectiveness of exercise training at once weekly on RCP may provide new insights in providing efficient a new training method for providing the improvement of aerobic capacity during high intensity exercise in the field of exercise physiology. Since such training results in specific physiological adaptations to high-intensity exercise that builds athletes’ high-intensity exercise tolerance, we hypothesized that low frequency severe-intensity interval training induces a marked shift of RCP toward high work rate during a ramp exercise test. The purpose of this study was to investigate the effect of low frequency severe-intensity interval training on RCP in ramp exercise test.

## Materials and Methods

### Subjects

Eighteen active male volunteers (age; 20.7 ± 2.2 years, range 18 to 29 years, height; 174.0 ± 5.6 cm, weight; 68.8 ± 13.5 kg) without cardiovascular risk factors participated in the study. The subject were recreational soccer players (*n* = 4), recreational runners (*n* = 4), recreational baseball player (*n* = 3), recreational basketball player (*n* = 2), recreational badminton player (*n* = 1), recreational volleyball player (*n* = 1), and recreational judoist (*n* = 3). All subjects were informed about the experimental procedures, potential risks and discomfort, and signed an informed consent form. The subjects of the present study included four subjects from our previous study (Nakahara et al., 2015). Participants were familiar with performing maximal bicycle ergometer exercise and the laboratory procedures for obtaining cardiorespiratory data. The protocol was submitted to and approved by the Ethical Committee of the Morinomiya University (No. 2014-035). All subjects gave written informed consent in accordance with the Declaration of Helsinki.

### Experimental Procedures and Protocols

Subjects were randomly assigned to one of the following two groups: low-frequency severe-intensity interval training (interval training group; *n* = 9), and no exercise training (control group; *n* = 9). Each subject underwent incremental maximal exercise tests before and after the interval training program described below. For 24 h preceding the day of each exercise test, the subjects were instructed to avoid strenuous exercise and to continue their usual diet. Food, alcohol and caffeine were prohibited from 4 h prior to each test.

### Exercise Training Program

The low-frequency severe-intensity interval training program was identical to that described in our previous study (Nakahara et al., 2015). Interval training was conducted at a frequency of one session per week for a duration of 3 months. The training program involved bicycle ergometer exercise. Each training session consisted of three bouts of cycling at 80% of maximum work rate. Each subject completed each bout until volitional fatigue. The recovery period between training bouts consisted of 2 min of active recovery by pedaling against no external resistance, and 1 min of rest. In the case that the exercise duration for the first bout was extended by 30% or more compared to first trial (first day of training) as an effect of the training program, then the training work rate for the next session (second stage) was increased by 10% from the initial setting. In each training session, all subjects were interviewed to ensure that they avoided strenuous exercise and had not changed their routine activities excluding training. On the other hand, the control group did not undertake formal training for any sports and their activity levels remained constant throughout the training period.

### Incremental Maximal Exercise Test

A computer-controlled bicycle ergometer (232CXL, Combi, Co., Tokyo, Japan) with an incremental ramp protocol was used to assess V̇_O__2_ peak, maximal work rate, VT, and RCP. Subjects were instructed to remain seated throughout the test and maintained the pedaling frequency of 60–70 rpm. The work rate was set at 20 W initially and increased by 1 W every 3 s (or 20 W per min) until the subject could no longer maintain a pedaling frequency of more than 50 rpm despite strong verbal encouragement. V̇_O__2_ peak and VT were determined by the V-slope method using the same criteria as in our previous study (Beaver et al., 1986; Wasserman et al., 1994; Keir et al., 2015). The criteria for the achievement of V̇_O__2_ peak were a plateau in O_2_ uptake (V̇_O__2_) despite increased work rate, and a respiratory exchange ratio above 1.10. The above-mentioned criteria are widely adopted in the literature of exercise physiology as primary validation for the attainment of V̇_O__2_ peak. RCP was defined using the criteria of increases in both V̇_E_/V̇_O__2_ and V̇_E_/V̇_CO__2_ and a decrease in end tidal carbon dioxide (PET_CO__2_) against V̇_O__2_ (Whipp et al., 1989; Oshima et al., 1997, 1998). The measurements were determined by visual inspection by two investigators. The ranges of isocapnic buffering and hypocapnic hyperventilation were defined as V̇_O__2_ from VT to RCP, and from RCP to the end of exercise, respectively.

### Experimental Apparatus and Measurements

Respiratory and metabolic data during the experiments were recorded using an automatic breath-by-breath gas analyzing system (ARCO2000-MET, ArcoSystem, Chiba, Japan) consisting of a differential pressure transducer, sampling tube, filter, suction pump and mass spectrometer. We recorded expired flow, CO_2_ and O_2_ concentrations at 200 Hz; and derived tidal volume, respiratory frequency, V̇_E_, PET_CO__2_, V̇_O__2_, and V̇_*C*__O__2_ from the digitized data. The gas analyzers were calibrated before each test. Heart rate was monitored via a three-lead electrocardiogram (BSM-7201, Nihon Kohden, Tokyo, Japan), and the beat-to-beat heart rate was recorded continuously using a personal computer in on-line mode at a sampling rate of 200 Hz during each test.

### Statistical Analysis

All data are expressed as mean ± standard deviation. Differences in all baseline physiological variables between groups were analyzed using unpaired Student’s *t*-test. Comparisons of variables before and after the training program, and between groups were performed by two-way ANOVA (group × time) with repeated measures. *Post hoc* analyses were conducted using Scheffe’s procedure. Statistical significance was defined at *p* < 0.05.

## Results

### Subjects and Training Program

Height and body weight before intervention were not significantly different between the interval training group (172.3 ± 3.9 cm and 70.9 ± 15.2 kg, respectively) and control group (175.7 ± 6.7 cm and 66.8 ± 12.0 kg). Relative value of V̇_O__2_ peak measured before intervention was also not significantly different between the training group (46.9 ± 4.9 ml/min/kg) and control group (48.6 ± 7.6 ml/min/kg).

In the interval training group (*n* = 9), the mean maximum exercise duration in first bout was extended from 401.0 ± 94.2 to 552.2 ± 134.1 s (39% increase, *p* = 0.001). On the other hand, the mean maximum exercise duration in the second bout (from 158.4 ± 52.1 to 158.9 ± 68.2 s) and in the third bout (from 153.2 ± 65.5 to 160.0 ± 57.9 s) was not extended.

### Maximal Exercise Stress Test

Figure 1 shows the breath-by-breath measurements of V̇_CO__2_, V̇_E_, V̇_E_/V̇_CO__2_, V̇_E_/V̇_O__2_, and PET_CO__2_ against V̇_O__2_ during the progressively increasing maximal exercise test of a representative subject in the interval training group, before (A; pretraining) and after the intervention program (B; post-training). Both before and after the intervention program, V̇_E_ and V̇_CO__2_ increased linearly with increase in V̇_O__2_ until VT and continued to increase slightly more steeply to RCP. PET_CO__2_ was maintained constant despite the increase in V̇_O__2_. Beyond the period of isocapnic buffering and RCP, V̇_E_ increased more steeply than V̇_CO__2_ increase, and was accompanied by a decrease in PET_CO__2_ and an increase in V̇_E_/V̇_CO__2_. The values of V̇_O__2_ and work rate at VT and RCP increased after the interval training program (B) compared to before the program (A).

Table 1 shows physiological parameters obtained during the maximal exercise stress test before and after the intervention program in the interval training (*n* = 9) and control (*n* = 9) groups. V̇_O__2_ peak was greater after intervention then before intervention.

**TABLE 1:** Comparison of pre- and post-intervention respiratory variables obtained during maximal exercise test in interval training group and control group.

	**Interval training**		**Control**	
	**group (*n* = 9)**		**group (*n* = 9)**	
	**Pre**	**Post**	**Pre**	**Post**
**Vo_2_ peak**				
(ml/min)	3279 ± 511	3698 ± 409***	3205.7 ± 483	3147.6 ± 347
	(2735–4385)	(3163–4633)	(2602–3760)	(2698–3706)
(ml/min/kg)	46.9 ± 4.9	52.6 ± 7.5**	48.6 ± 7.6	47.8 ± 7.6
	(37.0–51.6)	(42.5–59.1)	(38.0–57.8)	(33.0–55.3)
**V_E_ peak**				
(l/min)	153 ± 26	161 ± 28	158 ± 39	162 ± 31
**V_T_ peak**				
(ml)	2488 ± 307	2790 ± 483	2603 ± 307	2508 ± 208
**RR peak**				
(breaths/min)	62 ± 5	59 ± 13	61 ± 13	66 ± 14

Values are expressed as means ± SD. Values in parenthesses are ranges. VO_2_, oxygen uptake; VE, minute ventilation; VT; tidal volume; RR; respiratory rate. **p < 0.01; ***p < 0.001 significant difference within groups from pre- to post-training.

Table 2 shows mean cardiorespiratory variables at VT and at RCP, and the ranges of V̇_O__2_ for isocapnic buffering and hypocapnic hyperventilation obtained during the maximal exercise stress test before and after the intervention program in the interval training (*n* = 9) and control (*n* = 9) groups. In the interval training group, tidal volume and V̇_O__2_ at VT and at RCP were greater after intervention than before intervention.

**TABLE 2:** Comparison of pre- and post-intervention cardiorespiratory variables at the ventilatory threshold and the respiratory compensation point in interval training group and control group.

	**Interval training group (*n* = 9)**		**Control group (*n* = 9)**	
	**Pre**	**Post**	**Pre**	**Post**
**Ventilatory threshold**				
V_O__2_ (ml/min)	2153 ± 260	2542 ± 276***^##^	1925 ± 270	1921 ± 251
V_E_ (l/min)	64 ± 6	74 ± 10	67 ± 14	71 ± 14
V_T_ (ml)	2042 ± 251	2328 ± 420*	2004 ± 223	2022 ± 223
RR (breaths/min)	30 ± 5	32 ± 3	34 ± 5	37 ± 6
VE/VCO_2_	30 ± 3	29 ± 3	30 ± 3	32 ± 5
P_*ETCO2*_	46 ± 6	46 ± 5	45 ± 4	45 ± 5
HR (beats/min)	159 ± 9	165 ± 8	160 ± 15	164 ± 14
%V_O__2_ peak	66 ± 8	69 ± 5	60 ± 5	61 ± 5
**Respiratory compensation point**				
V_O__2_ (ml/min)	2817 ± 450	3216 ± 382*^#^	2743 ± 458	2613 ± 348
V_E_ (l/min)	100 ± 12	111 ± 15	105 ± 25	105 ± 28
V_T_ (ml)	2318 ± 291	2734 ± 411*	2370 ± 283	2409 ± 266
RR (breaths/min)	46 ± 6	41 ± 4	44 ± 6	45 ± 9
VE/VCO_2_	33 ± 4	31 ± 3	34 ± 4	35 ± 6
P_*ETCO2*_	44 ± 5	44 ± 4	42 ± 7	41 ± 6
HR (beats/min)	182 ± 5	183 ± 5	180 ± 9	183 ± 7
%Vo_2_ peak	86 ± 3	87 ± 4	85 ± 4	83 ± 5
**Isocapnic buffering**				
(ml/min)	944 ± 851	617 ± 300	818 ± 336	692 ± 281
**Hypocapnic hyperventilation**				
(ml/min)	462 ± 130	482 ± 154	485 ± 130	535 ± 152

Values are expressed as means ± SD. VO_2_, oxygen uptake; Vco_2_, carbon dioxide production, VE, minute ventilation; VT; tidal volume; RR; respiratory rate; PETCO2, end-tidal carbon dioxide pressure; HR, heart rate. *p < 0.05; ***p < 0.001 significant difference within groups from pre- to post- training. ^#^*p* < 0.05; ^##^*p* < 0.01 significant difference between interval training group vs. control group.

For V̇_O__2_ peak and mean cardiorespiratory variables at VT and at RCP, and the ranges of V̇_O__2_ for isocapnic buffering and hypocapnic hyperventilation, ANOVA revealed a significant group × training interaction for V̇_O__2_ peak (absolute V̇_O__2_ peak; *F*_1_,_16_ = 27.1, *p* < 0.001, $\eta_{\text{p}}^{2}$ = 0.628, relative V̇_O__2_ peak; *F*_1_,_16_ = 11.6, *p* = 0.004, $\eta_{\text{p}}^{2}$ = 0.421), V̇_O__2_ (VT; *F*_1_,_16_ = 49.9, *p* < 0.001, $\eta_{\text{p}}^{2}$ = 0.757, RCP; *F*_1_,_16_ = 19.6, *p* < 0.001, $\eta_{\text{p}}^{2}$ = 0.749) and tidal volume (VT; *F*_1_,_16_ = 4.6, *p* = 0.048, $\eta_{\text{p}}^{2}$ = 0.222, RCP; *F*_1_,_16_ = 6.4, *p* = 0.022, $\eta_{\text{p}}^{2}$ = 0.287) both at VT and RCP. These results indicated greater changes in these variables in the interval training than those in the control group. Analysis of the simple main effect also confirmed that the changes were due to the significant training effects in improve absolute V̇_O__2_ peak, relative V̇_O__2_ peak and tidal volume at VT and RCP in the training group (*p* < 0.05).

On the other hand, ANOVA detected no group × training interaction for isocapnic buffering, hypocapnic hyperventilation and other cardiorespiratory measurements. In the control group, no differences in all cardiorespiratory variables before and after the study period were observed.

**FIGURE 2:**
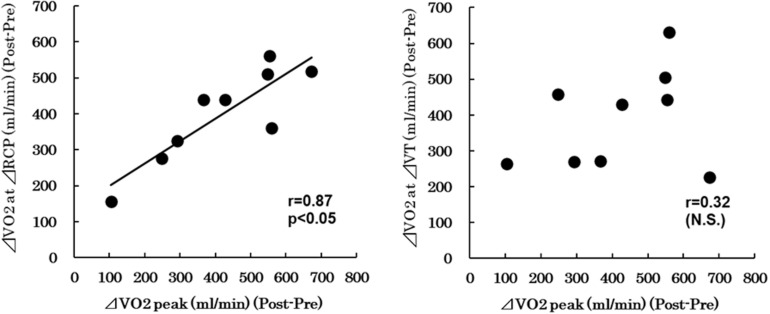
**(A)** Relationship between the change in V̇_O__2_ at RCP (ΔV̇_O__2_ at RCP = V̇_O__2_ at post-intervention RCP − V̇_O__2_ at pre-intervention RCP) and the change in V̇_O__2_ peak (ΔV̇_O__2_ peak = post-intervention V̇_O__2_ peak − pre-intervention V̇_O__2_ peak). **(B)** Relationship between the change in V̇_O__2_ at VT (ΔV̇_O__2_ at VT = V̇_O__2_ at post-intervention VT - V̇_O__2_ at pre-intervention VT) and ΔV̇_O__2_ peak. V_O__2_, oxygen uptake; VT, ventilatory threshold; RCP, respiratory compensation point.

Figure 2A shows the relationship between the change in V̇_O__2_ at RCP (ΔV̇_O__2_ at RCP = V̇_O__2_ at post-intervention RCP –V̇_O__2_ at pre-intervention RCP) and the change in V̇_O__2_ peak (ΔV̇_O__2_ peak = post-intervention V̇_O__2_ peak – pre-intervention V̇_O__2_ peak) obtained from individual subjects in the interval training group. A significant correlation between ΔV̇_O__2_ at RCP and ΔV̇_O__2_ peak (*r* = 0.87, *p* = 0.002) was observed. Figure 2B shows the relationship between the change in V̇_O__2_ at VT (ΔV̇_O__2_ at VT = V̇_O__2_ at post-intervention VT-V̇_O__2_ at pre-intervention VT) and ΔV̇O_2_ peak in individual subjects. No significant correlation between ΔV̇_O__2_ at VT and ΔV̇_O__2_ peak was found.

## Discussion

The present study demonstrated that severe-intensity interval training performed at a low frequency of once weekly markedly increased V̇_O__2_ at RCP by 15% during incremental exercise test, despite a lower training frequency than previous studies. In addition, the interval training changed the breathing pattern with higher tidal volume at high exercise intensities.

When expressed as percent V̇_O__2_ peak, RCP in the training group was 86% before intervention and 87% after intervention. These results are similar to those of previous studies (Loe et al., 2014; Oussaidene et al., 2015). Ciolac et al. (2011) assigned young college women to 40-min continuous training at an intensity of 70% V̇_O__2_ peak or 40-min interval training at 80–90% V̇O_2_ peak. The two training procedures were designed to have the same total training load and were conducted at a frequency of three times a week for 16 weeks. Comparison of the two groups showed that interval training (22.1%) was more effective than continuous training (8.8%) for improving V̇_O__2_ at RCP. Exceeding the RCP causes a number of metabolic changes in the body such as lactate accumulation in blood and blood pH reduction that leads to hyperventilation for the development of decompensated metabolic acidosis, thereby, limiting physiological performance (Maciejczyk et al., 2014b). These results suggest that low frequency severe-intensity interval training delays the onset of hyperventilation caused by respiratory compensation of metabolic acidosis during incremental exercise, indicating improvement in endurance performance at high intensity exercise. Ciolac et al. (2011) also suggested that the greater increase of submaximal aerobic capacity obtained high-intensity interval training could be caused by improvement of peripheral oxygen uptake such as a greater improvement on Ca^2+^cycling and mitochondrial capacity.

A positive correlation between ΔV̇_O__2_ peak and ΔRCP after the training program was also observed in the present study. On the other hand, there was no significant correlation between ΔV̇_O__2_ peak and ΔVT after the training program. Previous cross-sectional studies also indicated a positive correlation between V̇_O__2_ peak and RCP (Oshima et al., 1997; Takano, 2000). These results suggest that RCP can be a physiological indicator of the effectiveness of severe-intensity interval training program. Takano (2000) suggested that the correlation between V̇_O__2_ peak and RCP may in part explain why athletes can perform high-intensity exercise without excessive hyperventilation in response to metabolic acidosis. Iwaoka et al. also indicated that RCP and running velocity at RCP were strongly related to running times of 5000 and 10000 m in males (Iwaoka et al., 1988). Our results obtained from the present study support previous observations and extend previous knowledge. Despite a lower training frequency than previous studies, the exercise protocol in the present study achieved improvement of V̇_O__2_ at RCP. These results of minimum frequency severe-intensity interval training provide new insights into training programs for athletes, coaches, and athlete trainers.

Although the mechanisms by which severe-intensity interval training improves RCP are not completely elucidated in the study, the reduction of blood lactate concentration during high-intensity exercise may contribute to the improvement. Our previous study (2015) demonstrated that severe-intensity interval training induced an increase of exercise tolerance and a decrease of blood lactate concentration during constant load exercise at high-intensity. Meyer et al. (2004) observed a delay in RCP when blood acidosis is prevented by intravenous injection of bicarbonate, indicating that changes in blood pH are involved in the initiation of RCP. Hyperventilation elicited above the RCP induces a fall of arterial carbon dioxide tension, which is resulting from constraint of further falls of arterial pH due to severe lactic acidosis (Beaver et al., 1986; Wasserman et al., 1994). Therefore, a measurement of the blood lactate concentration could have been useful for the elucidation of mechanisms of a shift in RCP elicited by the interval training. In addition, the greater oxidative capacity in skeletal muscle may attribute to the improvement. Little et al. (2010) demonstrated that low-volume high-intensity interval training of six training sessions over 2 weeks effectively improved muscle mitochondrial capacity and exercise performance. Billat et al. (2003) also suggested that high-intensity interval training by running improves the aerobic potential of type IIA muscle, which in turn may become more fatigue-resistant. Our previous study confirmed that severe-intensity interval training once per week, which is identical to that described in the present study, resulted in increases of left ventricular mass and left ventricular posterior wall thickness measured at rest (Nakahara et al., 2015). Lucía et al. (1999a; 1999b; 2002) also speculated that greater ability of professional cyclists to tolerate at or above the RCP may be attributed to more efficient cardiac function, which is partially determined by greater myocardial wall, during high intensity exercise. Therefore, the cardiac morphological changes may also be related to improvement of RCP.

Interestingly, the present study indicated that tidal volume at RCP increased significantly after intervention compared to before intervention. In a cross-sectional study, Lucía et al. (1999b) investigated the breathing patterns of professional cyclists and amateur cyclists during incremental exercise test from submaximal to maximal intensities. They found no plateau in tidal volume at near-to maximal intensities during incremental exercise test in professional cyclists, and significantly higher tidal volume responses as a function of V̇_E_ during the exercise test in professional cyclists compared to amateur cyclists at higher exercise intensities. Takano (1993) indicated that tidal volume during the leg exercise was affected by mostly by metabolic variables such as V̇_CO__2_ and the respiratory rate was affected by the rate of limb movement. It has also been proposed that the change of tidal volume during exercise is more strongly associated by some markers of metabolic stimuli diving ventilation than respiratory rate. The response to respiratory rate during exercise is also contribute to various indicators including central command (Nicolò et al., 2017, 2018). Nicolò et al. (2018) indeed showed that respiratory frequency increased linearly with the augmentation in rating of perceived exertion, which is indirect evidence in central command, during exercise. Unfortunately, the present study was not measured the rating of perceived exertion during incremental exercise. As above mentioned, however, the results of study indicated that RCP was also 86% before intervention and 87% after intervention when expresses as percent V̇_O__2_ peak, suggesting that the activity of central command at RCP was possibly same between pre and post-training. In our study, the training achieved significant improvement of V̇_O__2_ at RCP and there was tendency for V̇_E_ at RCP to increase after intervention (*p* = 0.07). On the other hand, VE/VCO2 and P_*ETCO*__2_ at RCP did not change before and after intervention. Therefore, increased ventilation elicited by the increase of metabolic stimuli could be related to an increase in tidal volume at RCP after intervention. However, further research is required to elucidate the mechanisms of the increased tidal volume at RCP after intervention.

## Conclusion

We demonstrated that severe-intensity interval training once a week increased V̇_O__2_ at RCP during incremental exercise test. The results of minimum frequency severe-intensity interval training provide new insights into training program for athletes, coaches, and athlete trainers.

### Study Limitations

Some limitations of the present study should be mentioned. First, the training effects are strongly affected by the initial physical fitness level or training status of the subjects. The study was performed in untrained male with a high level of residual training plasticity, and it remains unclear whether the severe-intensity interval training can induce similar results in athletes. Second, the interval training was conducted for 3 months. Therefore, it remains unclear whether the effects of training can remain or increase further after 6–8 months of training at the same volume. Further study is needed to determine whether the once weekly interval training program can be used in athletes and be more effective in long term over 3 months.

## Data Availability Statement

All datasets generated for this study are included in the article/supplementary material.

## Ethics Statement

The studies involving human participants were reviewed and approved by the Ethical Committee of the Morinomiya University. Written informed consent to participate in this study was provided by the participants and the participants’ legal guardian/next of kin where necessary.

## Author Contributions

HN and TM designed the experiment and wrote the manuscript. All authors performed the analyses, interpreted the results, performed the experiments, discussed the results, and commented on the final manuscript.

## Conflict of Interest

The authors declare that the research was conducted in the absence of any commercial or financial relationships that could be construed as a potential conflict of interest.
